# Anti-inflammatory Properties of the Alpha-Melanocyte-Stimulating Hormone in Models of Granulomatous Inflammation

**DOI:** 10.1007/s00408-022-00546-x

**Published:** 2022-06-18

**Authors:** Abdolrazagh Hashemi Shahraki, Runxia Tian, Chongxu Zhang, Nevis L. Fregien, Pablo Bejarano, Mehdi Mirsaeidi

**Affiliations:** 1 Division of Pulmonary, Critical Care and Sleep, College of Medicine-Jacksonville, University of Florida, 655 West 11th Street, Jacksonville, FL 32209 USA; 2 Department of Cell Biology, University of Miami, Miami, FL USA; 3 Department of Pathology, Cleveland Clinic, Weston, FL USA

**Keywords:** Sarcoidosis, α-MSH, Bronchial epithelial cells, In vitro granuloma model, Mice model, Type I interferons

## Abstract

**Purpose:**

Alpha-melanocyte stimulating hormone (α-MSH) is known to have anti-inflammatory effects. However, the anti-inflammatory properties of α-MSH on normal bronchial epithelial cells are largely unknown, especially in the context of in vitro sarcoidosis models.

**Methods:**

We evaluated the anti-inflammatory effects of α-MSH on two different in vitro sarcoidosis models (lung-on-membrane model; LOMM and three-dimensional biochip pulmonary sarcoidosis model; 3D-BSGM) generated from NBECs and an in vivo sarcoidosis mouse model.

**Results:**

Treatment with α-MSH decreased inflammatory cytokine levels and downregulated type I interferon pathway genes and related proteins in LOMM and 3D-BSGM models. Treatment with α-MSH also significantly decreased macrophages and cytotoxic T-cells counts in a sarcoidosis mice model.

**Conclusion:**

Our results confirm the direct role of type I IFNs in the pathogenesis of sarcoid lung granulomas and highlight α-MSH as a potential novel therapeutic agent for treating pulmonary sarcoidosis.

**Graphical Abstract:**

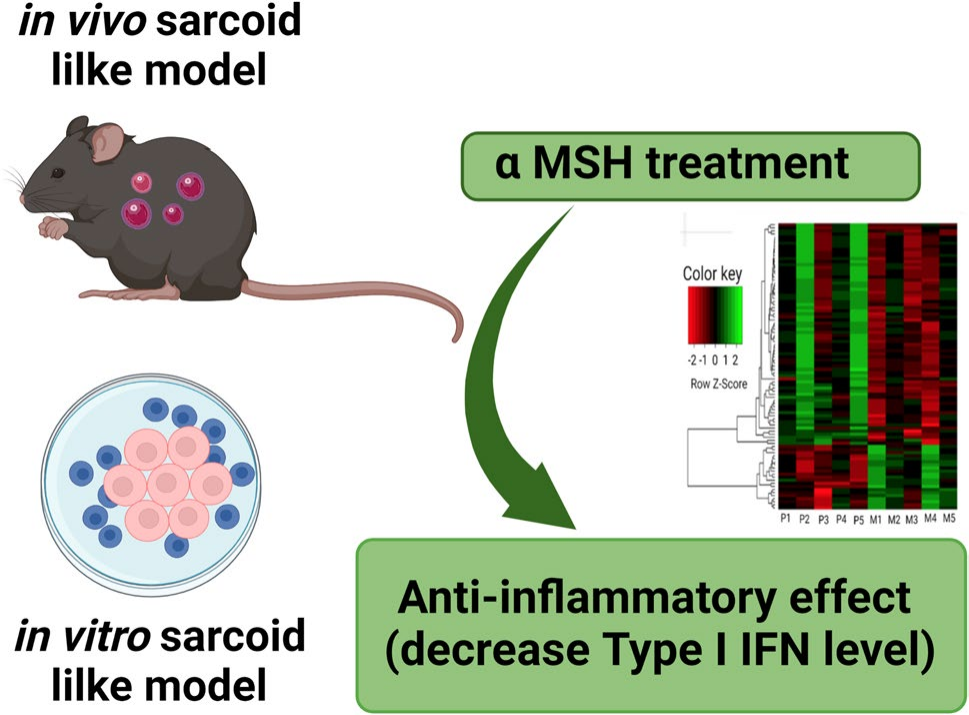

**Supplementary Information:**

The online version contains supplementary material available at 10.1007/s00408-022-00546-x.

## Introduction

Sarcoidosis is an inflammatory systemic and granulomatous disorder commonly affecting the lungs [1]. The incidence rate of sarcoidosis ranges from 7.6 to 8.8 per 100,000 per year in the United States (U.S.) [2], with a mortality rate between 2.8 and 7% [1, 3]. Corticosteroids such as prednisone remain the first choice therapy against symptomatic pulmonary sarcoidosis [4]. However, high doses of corticosteroids used for several months have deleterious side effects [5], so it is critical to evaluate other anti-inflammatory therapies.

Alpha-melanocyte-stimulating hormone (α-MSH) is an endogenous neuropeptide expressed in the pituitary gland and responsible for stimulating melanin production in hair and skin invertebrates. α-MSH has anti-inflammatory properties [6]. We recently developed an in vitro granuloma model by exposing peripheral blood mononuclear cells (PBMCs) from patients with sarcoidosis to microparticles generated from *the Mycobacterium abscessus* (MAB) cell wall [7]. The anti-inflammatory role of α-MSH on human bronchial epithelial cells (in vitro) or mice lung (in vivo) models has not been tested. We aimed to explore the anti-inflammatory effects of α-MSH treatment on in vitro and in vivo models. For in vitro studies, we developed a lung-on-membrane model (LOMM) using normal bronchial epithelial cells (NBECs) and a three-dimensional biochip for the pulmonary sarcoidosis model (3D-BSGM).

## Materials and Methods

### MAB Microparticle Production

MAB (isolate # CCUG 47942, gift from Dr. Malin Ridell, University of Gothenburg, Sweden) was used to isolate the cell wall microparticles as described previously by our team [8]. We used 100 μL of MAB microparticles diluted to a concentration equal to a multiplicity of infection (whole bacterium) of 10:1 to treat the developed lung models (bronchial epithelial cells side). Supernatant and cells were harvested 48 h after exposure for different analysis fields[8].

### Lung-on-Membrane Model (LOMM) Developed from NBECs

Our LOMM contains NBECs collected from 5 healthy donors (Supplementary Table 1). We generated the lung models as we described previously in [8] by culturing five × 10^5^ cells NBECs of each donor (5 donors × 3 replicates for each donor = 15 experiments) on the top side of transwell® polyester membrane cell culture inserts and human endothelial cells (2 × 10^5^ cells, Human Lung Microvascular Endothelial Cells, Lonza, Walkersville, MD) on the bottom side at the air–liquid interface (ALI). The LOMM models were split into a control (unchallenged group), challenged LOMM (challenged with MAB microparticle), and challenged LOMM + α-MSH (challenged with MAB microparticle and treated with 5 μM α-MSH (Bachem Americas, Inc, CA USA) every 24 h. In total, five LOMM were included in each group. In addition, one group received α-MSH (not challenged with MAB microparticles) and was another control, only for western blotting analysis of type I IFN proteins (MX1, OSA1, and ISG15). The cell culture media of all wells were collected after 48 h for measurements.

### Development of a Novel Three-Dimensional Biochip Pulmonary Sarcoidosis Model (3D-BSGM)

PBMCs were isolated from blood samples of 5 healthy donors, as we described before [7]. In the meantime, we also developed ALI from NBECs and human endothelial cells as described in the LOMM development above [7]. A little scratch was made on the ALI of the LOMM, and then 50 μL of the developed granulomas from PBMCs (2 × 10^6^ cells) were added to ALI on the microchip to develop 3D-BSGM as described elsewhere [9]. We designed 3 groups for this experiment: control (LOMM + unchallenged PBMCs), 3D-BSGM (LOMM + challenged PBMCs + saline as treatment), and 3D-BSGM + α-MSH (LOMM + challenged PBMCs + α-MSH as treatment). We used NBECs collected from 5 donors to develop LOMM and 3D-BSGM (Supplementary Table 1) with two replicates for each donor (2 replicates x NBECs from 5 donors × 3 groups = 30 experiments).

### Sarcoidosis Mouse Model

As previously described, we developed a mice granuloma model using MAB microparticles [10]. We grouped mice (C57Bl/6 male mice, eight weeks old, The Jackson Laboratory, ME, USA) into three different groups: control (challenged with saline), granuloma group (challenged with MAB microparticles and treated with saline), and granuloma + α-MSH group (challenged with MAB microparticles and treated with α-MSH; daily subcutaneously injections of 10 µg). Each group had four replicates (in total, 12 mice). MAB microparticles were administered intratracheally to the experimental group. The first dose was 5 × 10^8^ CFU in 50 µL. The three subsequent doses were 2 × 10^8^ CFU in 20 µL. The control group received only 20 µL of saline. The granuloma + α-MSH group also received α-MSH (10 µg) subcutaneously daily as treatment, while the granuloma and control group received saline. Mice were euthanized on day 21. We collected their left lungs for pathology after removing blood. We used H&E staining to determine inflammatory pathology [11]. For IHC, we used primary antibodies (CD68, PD-1, PD-L1, CD30) and secondary antibodies, all purchased from Cell Signaling Technology, Beverly, MA, USA [11]. ABC Elite kit (Cat# PK-6200 Vector Laboratories, Inc. Burlingame, CA, USA) was used to detect immunoreaction. We used the CD30 marker to measure lymphocyte activation [12], CD68 to quantify macrophages [13], and PD1 and PD-L1 markers to evaluate granuloma formation [14]. A pathologist evaluated three fields (100X power magnification) to score lung inflammation, as we reported before [11].

### ELISA for Measuring Cytokine Expression

The levels of cytokines (IL-1RA, IL-10, CCL2, IFNα, IFNγ, GM-CSF, and TNFα; purchased from Abcam, Cambridge, USA) were measured and compared in media collected from the LOMM models. For mature IL-1β, we used the IL-1 beta Human ELISA Kit (Thermo Fisher Scientific, USA, Cat# BMS224-2). We also measure the cytokine level in media collected from the 3D-BSGM models per the manufacturers' instructions.

### RNAseq and Pathway Analysis

Total RNA was extracted from the collected cells from 3D-BSGM models (treated with α-MSH and not treated with α-MSH) separately, as we described previously in [7, 8]. Almost 40 million single-end 75 base reads per sample were used for differential expression analysis using EdgeR software [15] with a false discovery rate p-value (FDR) ≤ 0.05. Enrichr online [16] and DAVID bioinformatics resource [17] were used for pathway enrichment analyses and to obtain the enriched biological processes (B.P.s), respectively. STRING version 11 [18] also was used for protein–protein interaction (PPI) network analysis of the dysregulated genes in the 3D-BSGM Model treated with α-MSH.

### Western Blotting

Cells were collected from LOMM and 3D-BSGM models separately and performed western blotting as described before [7, 8]. For LOMM, the PVDF membrane was probed with primary antibodies against 2′-5′-Oligoadenylate Synthetase 1 (OAS1) Rabbit mAb, Interferon-induced GTP-binding protein Mx1 (MX1) Rabbit mAb, and Interferon-stimulated gene 15 (ISG15) Rabbit mAb. For 3D-BSGM, the PVDF membrane was sequentially probed with primary antibodies against ISG15 Rabbit mAb, IL-2 Rabbit mAb, IFN-α Rabbit mAb, p-NF-kB Rabbit mAb, and NF-kB Rabbit mAb. All antibodies were purchased from Proteintech Group, Inc. Rosemont, IL, USA. After adding horseradish peroxidase-conjugated goat anti-rabbit antibody, the secondary antibodies were detected using enhanced chemiluminescence. We used beta-actin as a control.

### Flow Cytometry

For each lung sample, a single-cell suspension was prepared by [19]. The cells (10^6^ cells mL^−1^) were resuspended in 100 μL protein blocking solution with five μL fluorescent-conjugated antibodies against CD3, CD4, CD8, CD45, CD68, and PD-L1, Siglec F, CD11b, CD11c, CD64, MHC II, Ly6Clo, CD103, and CD24; all purchased for Abcam, Cambridge, USA. Samples were analyzed on a BD LSR II flow cytometer using BD FACSDiva software, and data analysis was performed using Flowjo software (TreeStar, Ashland, OR, USA). Cell populations were identified using a sequential gating strategy. We also measured the intracellular IFNγ in lung cells using flow cytometry and intracellular staining of IFNγ by Cyto-Fast™ Fix/Perm Buffer Set (BioLegend, USA).

We separated alveolar macrophage (Siglec F^+^CD11b^−^CD11c^+^CD64^+^) from monocytes/undifferentiated macrophages (CD11b^+^ MHC II^−^CD64^+/−^Ly6Clo^+^) as described before [20]. DCs were sorted and defined as CD11c^+^CD103^+^CD24^+^ [20]. CD45-expressed cells were sorted using flow cytometry [21]. CD68 (a pan-macrophage marker) was used to classify leukocyte subpopulations of mice lungs into three groups: CD68 negative (CD68^−^), CD68 low, and CD68 high [21].

### Statistical Analysis

One-way analysis of variance (ANOVA) and nonparametric analysis (Friedman and Dunn's multiple comparison tests) were used to compare the variation of different cytokine levels among groups using GraphPad Prism 8 software. Data were corrected for multiple comparisons using Dunnett correction. Data represent the mean ± SEM of the replicates. *Our analysis defined a P-*value (two-sided) less than 0.05 as statistically significant.

## Results

### α-MSH Has Anti-inflammatory Effects in an In Vitro Human Lung-on-Membrane Model (LOMM)

**Fig. 1:**
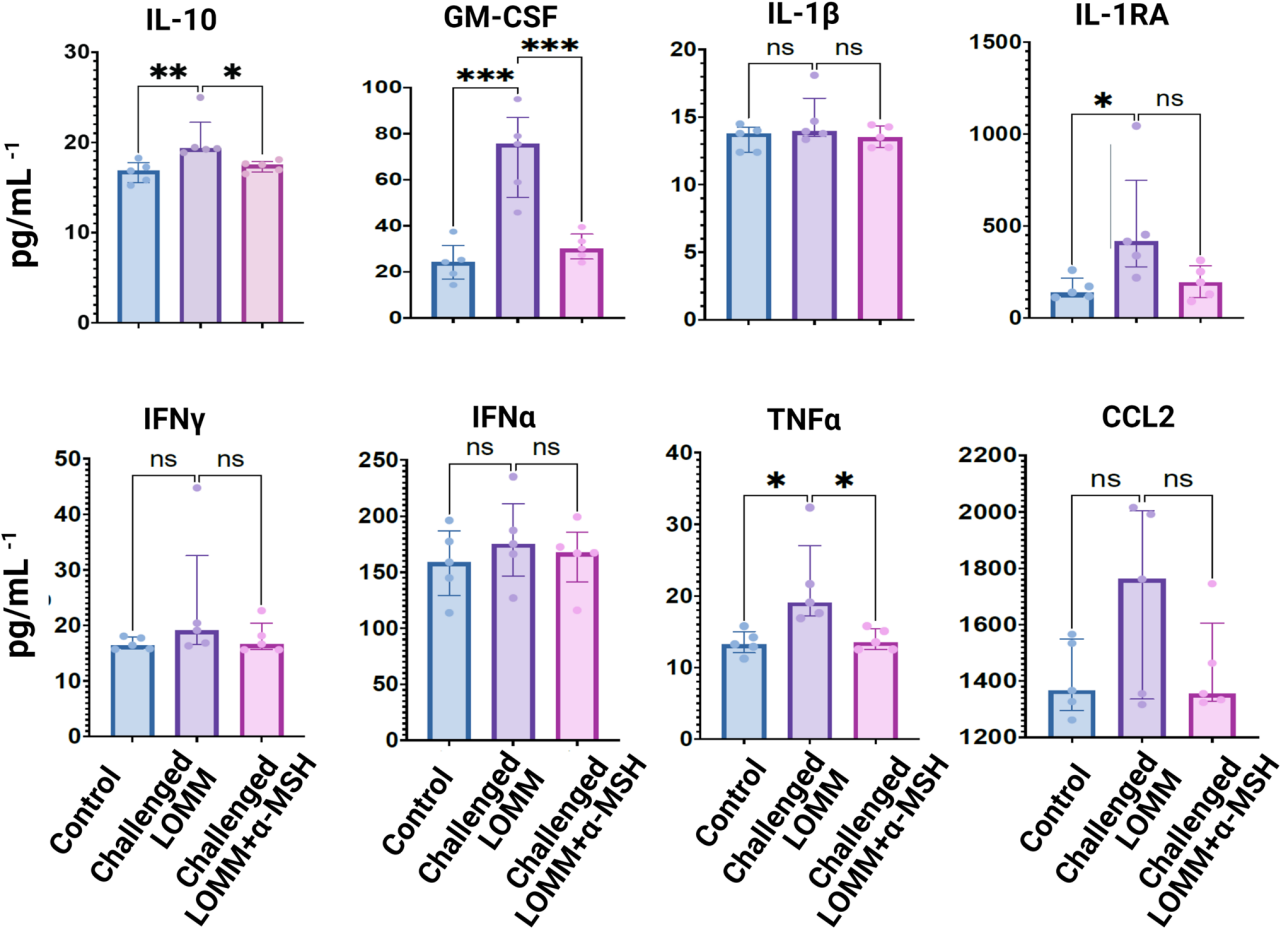
Shows the level of different cytokines measured from 3 different LOMM, including Control, Challenged LOMM, and Challenged LOMM + α-MSH. Two groups of LOMM were challenged with MAB microparticles (Challenged LOMM and Challenged LOMM + α-MSH). The challenged LOMM model received saline as treatment while the Challenged LOMM + α-MSH received α-MSH as treatment. Each LOMM was generated from the NBECs of 5 different donors, and each donor had three replicates. The mean value of the replicates (*n* = 3) for each donor was used to generate the graphs. In total, five LOMM were included in each group—the mean value of replicates. Significant variations are highlighted for each plot. ns: no significant variation; *indicates significant variations (*: < 0.05, ** < 0.005 and ***: < 0.0005)

We hypothesized that α-MSH downregulates inflammatory cytokines in LOMM after challenge with MAB microparticles. LOMM was developed as presented in [8] using NBECs (Fig. S1). The MAB particle challenged LOMM model showed increases in all measured cytokines and statistically significant increases in GM-CSF, IL-1RA, IL-10, and TNFα compared to the control. There were numeric reductions in all measured cytokine concentrations after α-MSH treatment (challenged LOMM + α-MSH), with a statistically significant decrease in GM-CSF, IL-10, and TNFα concentration in comparison to the challenged LOMM not treated with α-MSH (Fig. 1).

### Anti-inflammatory Effect of α-MSH Through IFN Type I in LOMM Model

**Fig. 2:**
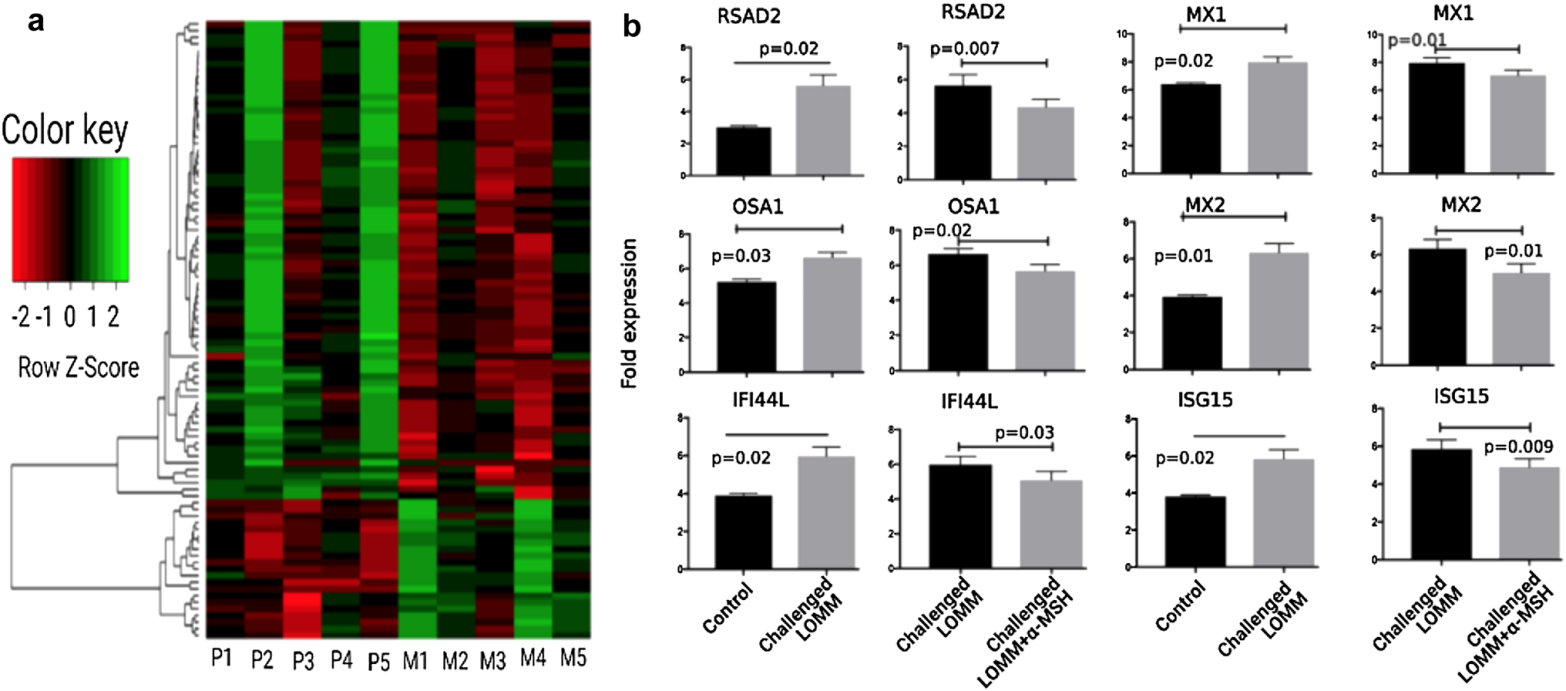
Heat map of RNA-Seq transcriptome analysis of challenged LOMM (P1-5; no α-MSH treatment) and challenged LOMM + α-MSH (M1-5; after α-MSH treatment). **a** Genes with significant variation in their expression level between two groups (> 2.5-fold increase; green or decrease; red) are highlighted. The sample size was 5 for each group. **b** Bar graphs also show significant changes in type I IFN genes among three LOMM groups; control, challenged, and challenged + α-MSH

**Fig. 3:**
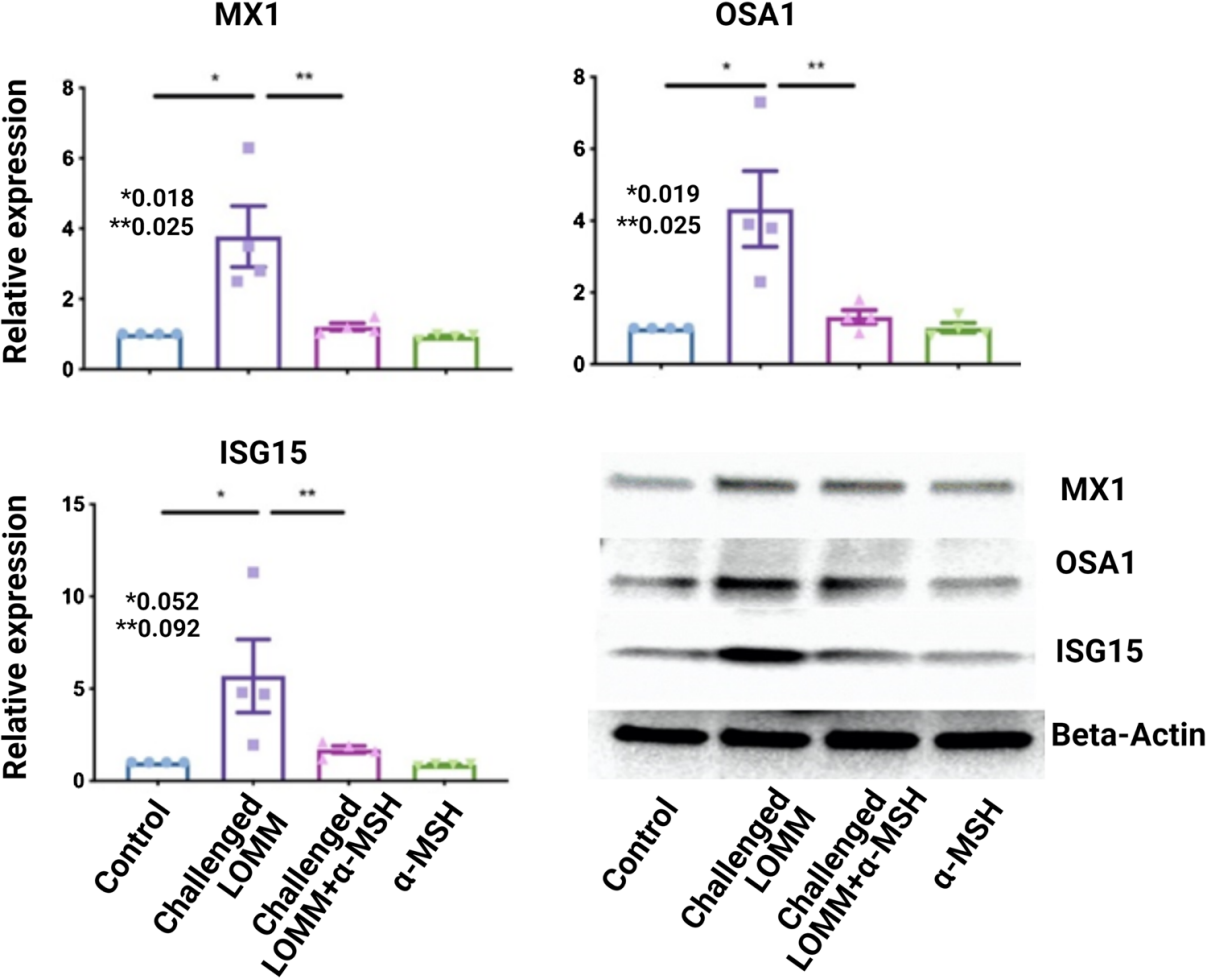
Type I IFN proteins (MX1, OSA1, and ISG15) were expressed in 4 LOMM groups; control challenged LOMM and LOMM + α-MSH and α-MSH only. In total, four LOMM (from four different human subjects) were included in each group. Only two groups, including challenged LOMM and challenged LOMM + α-MSH, were exposed to MAB microparticles. Control received only saline, while the α-MSH group was only treated with α-MSH and were used as controls for this experiment. Significant variations are highlighted for each plot

We hypothesized that α-MSH downregulates type I IFN-induced genes and proteins in LOMM after a challenge with MAB microparticles. Transcriptomic analysis (Fig. 2a) showed a significant reduction in class I IFN pathway genes after treatment with α-MSH. To further test the Effect of α-MSH on type I IFN protein expression, we measured the relative expressions of RSAD2, MX1, MX2, OSA1, IFI44L, and ISG15 proteins as we observed a significant variation of type I IFN proteins encoding genes (Fig. 2b). Relative expressions of MX1, OSA1, and ISG15 proteins in western blotting were significantly decreased in the challenged LOMM + α-MSH group compared to the challenged LOMM group, as shown in Fig. 3.

### α-MSH Has Anti-inflammatory Effects in an In Vitro Novel Three-Dimensional Biochip Pulmonary Sarcoidosis Model (3D-BSGM)

The 3D-BSGM Model [9] was used to test the hypothesis that α-MSH reduces inflammatory cytokines in an integrated granuloma model with a lung biochip. Our previous study developed a new integrated LOMM and granuloma called 3D-BSGM using PMBCs exposed to MAB microparticles to create a human sarcoid-like granulomas Field [9] effectively. This study used MAB microparticles to develop 3D-BSGM human sarcoid-like granulomas (Fig. S2a). The anti-inflammatory properties of α-MSH were proven in this complex Model, with significant reduction of IL-1β and GM-CSF measured from the culture media of the α-MSH-treated group (3D-BSGM + α-MSH) in comparison to 3D-BSGM as shown in Fig. S2b.

**Fig. 4:**
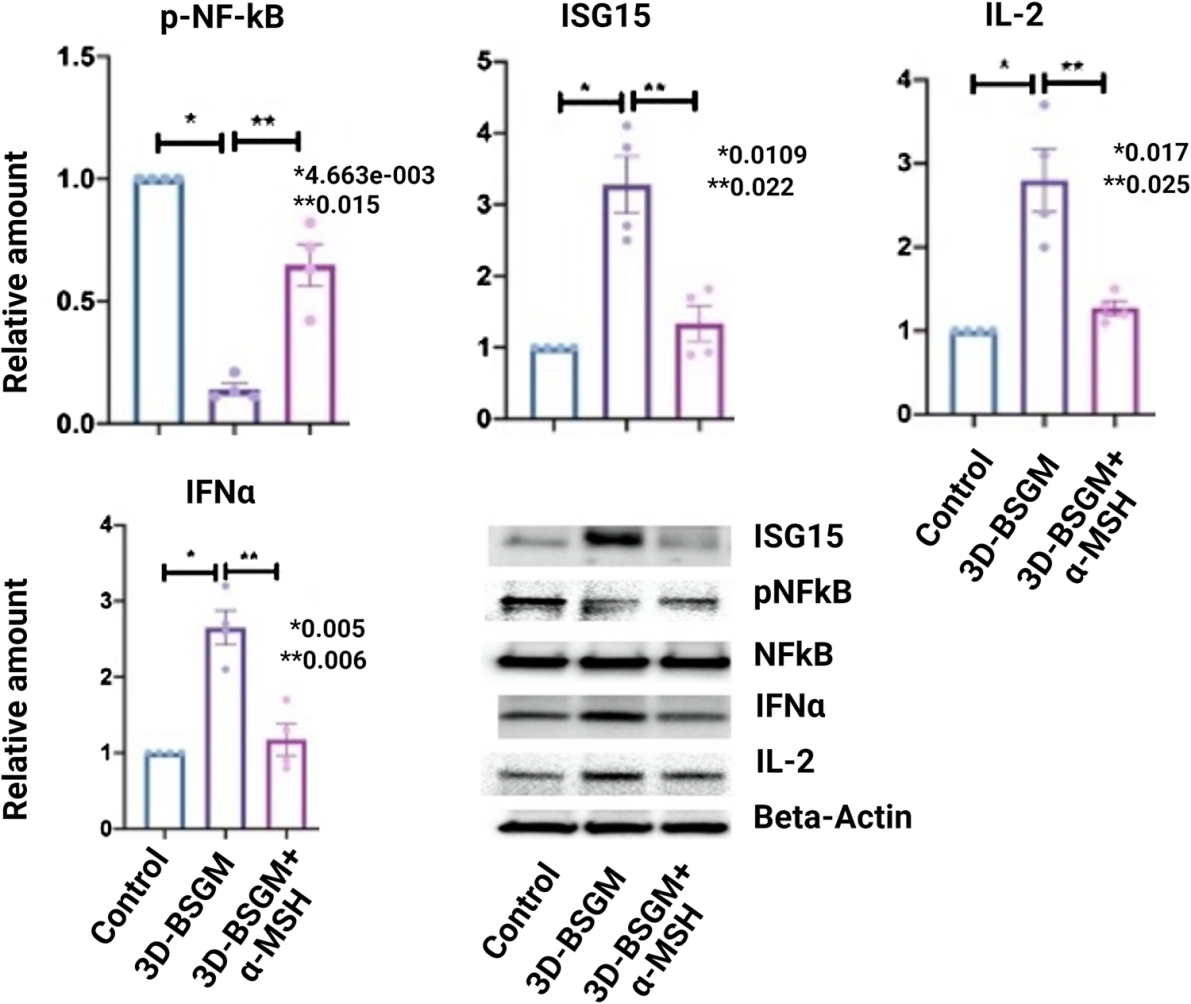
Shows representative western blotting for three groups: control, 3D-BSGM, and 3D-BSGM + α-MSH. Two 3D-BSGM groups were challenged with MAB microparticles to generate granuloma, and a group only received saline to serve as the control. In addition, one of the 3D-BSGM groups was treated with α-MSH, but the other only received saline as treatment. Four 3D-BSGM were included in each group, generated from 4 different donors. Significant variations are highlighted for each plot

To test the protein expression of cells in the integrated granuloma model (3D-BSGM), we performed western blotting after protein extraction from each chip. As shown in Fig. 4, the type I IFN cytokines (ISG15 and IFNα) expression increased in the granuloma model (3D-BSGM) but reduced in the granuloma model after α-MSH treatment (3D-BSGM + α-MSH). Similarly, IL-2 expression increased in the 3D-BSGM, while treatment with α-MSH significantly reduced its expression in the 3D-BSGM + α-MSH group. In addition, activated NF-kB (p-NF-kB) expression was decreased in 3D-BSGM compared to the control group but normalized in the 3D-BSGM + α-MSH group.

### Transcriptomics Changes in the 3D-BSGM Model After α-MSH Treatment

We found that 89 genes were upregulated in 3D-BSGM without α-MSH treatment, while only 11 remained upregulated after treatment. Notably, we found that AQP9 (encoding of aquaporin-9), MARCO (encoding of macrophage receptor MARCO), and CD53 (encoding of leukocyte surface antigen CD53) were upregulated in 3D-BSGM (not treated α-MSH); however, their expression was downregulated in the 3D-BSGM after α-MSH treatment. Protein–protein interaction network analysis of the top 68 dysregulated genes in 3D-BSGM treated with α-MSH and biological process affected after α-MSH treatment are presented in Fig.S3 and S4, respectively. Our analysis revealed 95 unique genes were downregulated after using α-MSH treatment in 3D-BSGM (Fig.S5).

### α-MSH Effects in a Sarcoidosis Mice Model

**Fig. 5:**
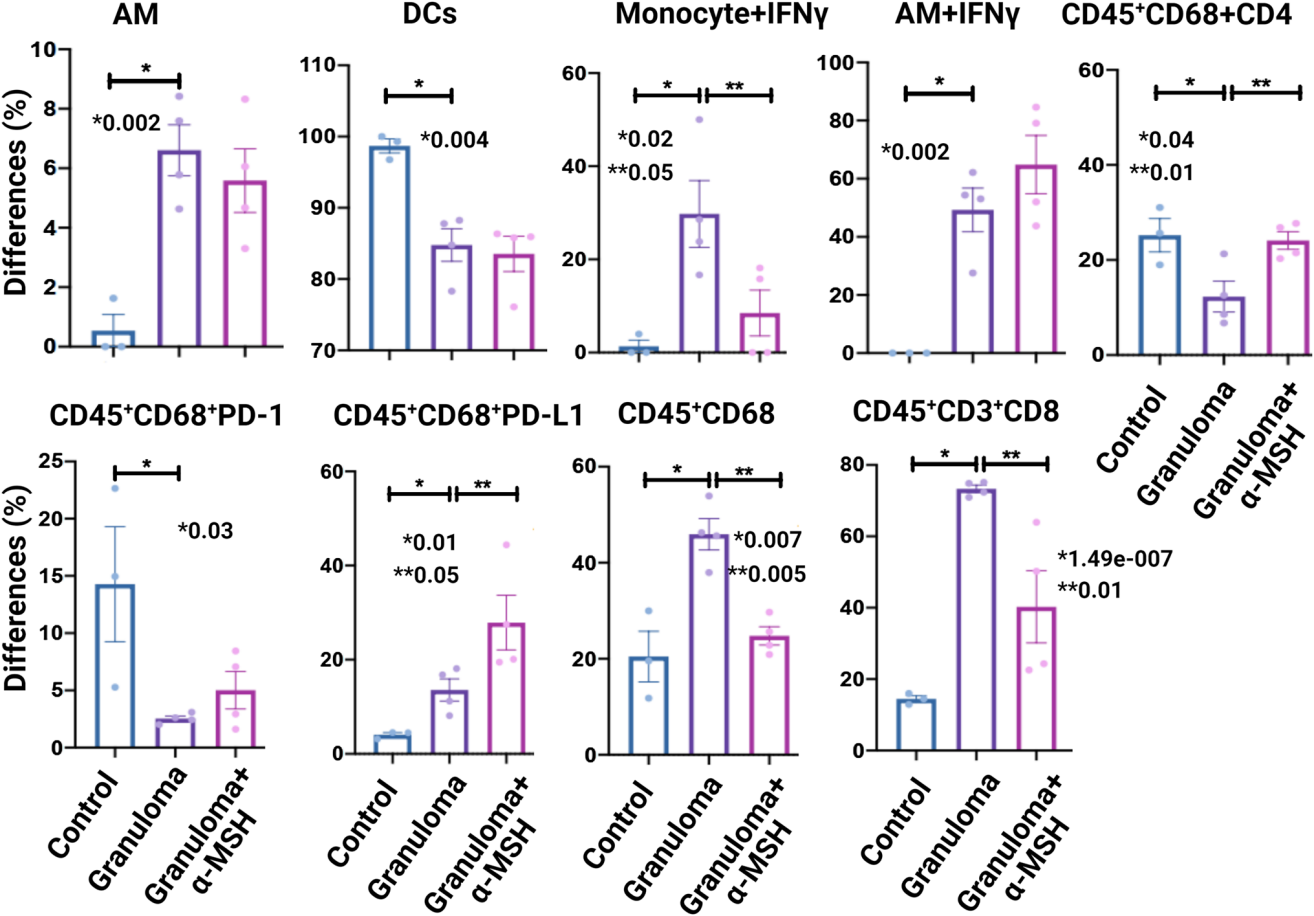
Flow cytometry results of isolated single pulmonary cells from 3 groups of mice, control, granuloma (challenged with MAB microparticles and treated with saline), and granuloma + α-MSH (challenged with MAB microparticles and treated with daily α-MSH). *AM* alveolar macrophage, *DCS* dendritic cells. Each Model had four mice. Alveolar macrophage and monocytes with internalized IFNγ were also presented. Siglec F^+^ CD11b^−^ CD11c^+^ CD64^+^ cells were identified as alveolar macrophage while CD11b^+^ MHC II^−^ CD64^+/−^ Ly6Clo^+^ were grouped as monocytes/undifferentiated. DCs were defined as CD11c^+^ CD103^+^ CD24^+^. Significant variations are highlighted for each plot

We developed mice granuloma models using the MAB microparticles as previously described (Fig. S6a) [10] to test in vivo effects of α-MSH. Mice were exposed to MAB microparticles via intratracheal injection to develop granuloma. The mice were grouped as control, granuloma, and granuloma + α-MSH. The lungs were removed after three weeks. Lungs were fixed and stained for CD30, CD68, PD-1, and PD-L1 (Fig.S6b). In addition, flow cytometry was performed on single lung cells to evaluate the effects of α-MSH on the immune response of the sarcoidosis mice model (Fig. 5)*. *In vivo analysis of α-MSH-treated granulomas in sarcoidosis mice model revealed a numerical decrease in all measured leukocytes and cytokines, with a statistically significant reduction in CD45^+^CD68 and CD45^+^CD3^+^CD8 in granuloma + α-MSH group relative to the granuloma group.

## Discussion

Granulomatous inflammation in sarcoidosis is believed to be caused by a persistent, poorly degradable unknown antigen, including mycobacterial antigens, combined with a non-resolving host response. As the pathogenesis of sarcoidosis is not well known, there is no perfect model to study sarcoidosis. Therefore, we used MAB microparticles as an antigen to stimulate the immune system and generate granulomas. As we are using the cell wall of opportunistic mycobacteria (MAB), the generated granuloma is less caseous than the tuberculosis granuloma and is more like sarcoid granuloma. The same concept was suggested by Locke and co-workers [22] to develop a sarcoid granuloma.

Inflammatory cytokines and interferon pathways and proteins decreased after treatment with α-MSH in both LOMM and 3D-BSGM, indicating the anti-inflammatory effect of α-MSH; however, we did not measure inflammatory cytokines and interferon pathways in the mice model which is a limitation of current research. α-MSH significantly reduces the inflammatory response in bronchial epithelial cells challenged with MAB microparticles. α-MSH treatment also reduces Type I IFN protein expressions. α-MSH also shows solid anti-inflammatory properties in a complex multilayer in vitro model. α-MSH treatment in a mice pulmonary granulomatous model resulted in a statistically significant decrease in inflammatory cell numbers, including CD45^+^CD68 expression (macrophages) and CD45^+^CD3^+^CD8 expression (cytotoxic T-cells).

α-MSH has an anti-inflammatory effect at different layers by downstream inhibition of NF*-*kB nuclear translocation [23] or through the calcium signaling pathway [24] or mTOR signal pathway [25]. Our previous study on PBMCs showed that α-MSH anti-inflammatory properties are associated with the increasing competition of phosphorylated CREB (p-CREB) with NF*-*kB to sit on promotors of pro-inflammatory genes, including *IL-2*, *IL-6*, *IL7*, *IL-10*, *TNFα*, and *IFNγ* [7]*.* p-CREB inhibits phosphorylated NF-kB (p-NF*-*kB) bounds to the promotor regions of cytokine genes, thereby limiting pro-inflammatory responses [26]. In the present study, we observed an anti-inflammatory effect of α-MSH through increases in the level of p-NF*-*kB. Decreased type I IFNs and pro-inflammatory cytokines were consistent with our previous study on PBMCs in the granulomatous inflammation [7]. Type I IFNs (IFNα and IFNβ) are different from IFNγ, a commonly cited cytokine implicated in the granuloma formation [27]. IFNα increases the expression of MHC class II antigens and the expression of IL-12, which work to maintain the lymphocytic Th1 response in granulomatous inflammation [28]. Type I IFNs-mediated granuloma formation likely has a high prevalence in the lung, secondary to the localized expressions of both β1 and β2 subunits of IL-12R in the lung seen on bronchoalveolar lavage [29–31].

In vivo mice, the lung model showed decreases in leukocytes CD45^+^CD68 (macrophages) and CD45^+^CD3^+^CD8 (cytotoxic T-cells) after treatment with α-MSH. Macrophages play an essential role in granulomatous inflammation; they release pro-inflammatory cytokines to induce T-cell activity. IFNγ, produced by Th cells, activates macrophages (M1 subtype). Activated macrophages fuse into phagocytic-multinucleated epithelioid cells to form granulomas maintained by TNFα [32]. It has been reported that α‐MSH suppresses CD14 expression [33] and blocks TLR4 signaling, which could suppress macrophage activities after LPS stimulation. In addition, α‐MSH suppresses macrophages through TLR4 [34] or by blocking the activation of p38 MAPK [35] and NF‐κB [36]. Our results show, for the first time, that α‐MSH can decrease the number of macrophages in sarcoidosis granuloma.

Our transcriptome analysis revealed that AQP9, CD53, and MARCO levels increased in 3D-BSGM (not treated α-MSH) after challenging the cells with MAB particles while treating the cells with α-MSH decreased the AQP9, CD53, and MARCO levels indicating the anti-inflammatory property of α-MSH. AQP9 is the central glycerol channel that may play a role in specialized leukocyte functions such as immunological response to inflammation [37]. CD53 is also critical in immune cell adhesion and migration during inflammation [38]. In addition, MARCO can be upregulated on macrophages after bacterial infection suggesting its role in removing pathogens [39].

Very few studies have explored the role of cytotoxic T-cells, while the role of helper T-cells is well known in sarcoidosis [40]. Cytotoxic T-cells are mainly involved in granule-mediated lysis of altered or infected cells by releasing cytolytic effector molecules (granzyme A and B) and antimicrobial peptides [41]. Parasa et al. found higher proportions of peripheral cytotoxic T-cells expressing perforin and granzyme B (higher level of cytotoxicity) in sarcoidosis patients compared to healthy controls [42]. Our data confirmed that α‐MSH could decrease the number of cytotoxic T-cells in sarcoidosis. Our results show that a α-MSH-mediated reduction in type I IFNs in vitro modeling correlates with the macrophage and cytotoxic T-cell reduction seen in *vivo* modeling. With minor type I IFN signaling, there is less activation of helper T-cells and subsequently less IFNγ-mediated activation of macrophages. Similarly, with minor type I IFNs signaling, there is less direct activation of cytotoxic T-cells, the mechanism usually responsible for cell lysis.

In conclusion, we have demonstrated that α-MSH is an anti-inflammatory effector. Our data also shows that α-MSH could be considered a new potential therapy for treating pulmonary sarcoidosis via modulation of pro-inflammatory cytokines and immune cells in the granuloma.

## Supplementary Information

Below is the link to the electronic supplementary material.

Supplementary file. (DOCX 4859 KB)

## Data Availability

Data are contained within the article and supplementary material.

## References

[1] Mirsaeidi M, Machado RF, Schraufnagel D, Sweiss NJ, Baughman RP (2015). Racial difference in sarcoidosis mortality in the United States. Chest.

[2] Baughman RP, Field S, Costabel U, Crystal RG, Culver DA, Drent M (2016). Sarcoidosis in America: analysis based on health care use. Ann Am Thorac Soc.

[3] Jamilloux Y, Valeyre D, Lortholary O, Bernard C, Kerever S, Lelievre L (2015). The spectrum of opportunistic diseases complicating sarcoidosis. Autoimmun Rev.

[4] Schutt AC, Bullington WM, Judson MA (2010). Pharmacotherapy for pulmonary sarcoidosis: a Delphi consensus study. Respir Med.

[5] Paramothayan NS, Lasserson TJ (2005). Jones P (2005) Corticosteroids for pulmonary sarcoidosis. Cochrane Database Syst Rev.

[6] Mykicki N, Herrmann AM, Schwab N, Deenen R, Sparwasser T, Limmer A (2016). Melanocortin-1 receptor activation is neuroprotective in mouse models of neuroinflammatory disease. Sci Transl Med.

[7] Zhang C, Chery S, Lazerson A, Altman NH, Jackson R, Holt G (2020). Anti-inflammatory effects of α-MSH through p-CREB expression in sarcoidosis like granuloma model. Sci Rep.

[8] Zhang C, Asif H, Holt GE, Griswold AJ, Campos M, Bejarano P (2019). Mycobacterium abscessus—bronchial epithelial cells crosstalk through type I interferon signaling. Front Immunol.

[9] Calcagno TM, Zhang C, Tian R, Ebrahimi B, Mirsaeidi M (2021). Novel three-dimensional biochip pulmonary sarcoidosis model. PLoS ONE.

[10] Urdaneta G, Zhang C, Tian R, Bejarano P, Schally A, Holt G (2020). A novel therapeutic effect of MIA-602 presented in an in vivo sarcoidosis mouse model. Am J Respir Crit Care Med.

[11] Zhang C, Tian R, Dreifus EM, Hashemi Shahraki A, Holt G, Cai R (2021). Activity of the growth hormone-releasing hormone antagonist MIA602 and its underlying mechanisms of action in sarcoidosis-like granuloma. Clin Transl Immunol.

[12] Ahmad S, Azid NA, Boer JC, Lim J, Chen X, Plebanski M (2018). The key role of TNF-TNFR2 interactions in the modulation of allergic inflammation: a review. Front Immunol.

[13] Francisco LM, Sage PT, Sharpe AH (2010). The PD-1 pathway in tolerance and autoimmunity. Immunol Rev.

[14] Braun NA, Celada LJ, Herazo-Maya JD, Abraham S, Shaginurova G, Sevin CM (2014). Blockade of the programmed death-1 pathway restores sarcoidosis CD4+ T-cell proliferative capacity. Am J Respir Crit.

[15] Robinson MD, McCarthy DJ, Smyth GK (2010). edgeR: a Bioconductor package for differential expression analysis of digital gene expression data. Bioinformatics.

[16] Chen EY, Tan CM, Kou Y, Duan Q, Wang Z, Meirelles GV (2013). Enrichr: interactive and collaborative HTML5 gene list enrichment analysis tool. BMC Bioinform.

[17] Sherman BT, Lempicki RA (2009). Systematic and integrative analysis of large gene lists using DAVID bioinformatics resources. Nat Protoc.

[18] Szklarczyk D, Gable AL, Lyon D, Junge A, Wyder S, Huerta-Cepas J (2019). STRING v11: protein-protein association networks with increased coverage, supporting functional discovery in genome-wide experimental datasets. Nucleic Acids Res.

[19] Pösel C, Möller K, Boltze J, Wagner D-C, Weise G (2016). Isolation and flow cytometric analysis of immune cells from the ischemic mouse brain. J Vis Exp.

[20] Misharin AV, Morales-Nebreda L, Mutlu GM, Budinger GS, Perlman H (2013). Flow cytometric analysis of macrophages and dendritic cell subsets in the mouse lung. Am J Respir Cell Mol.

[21] Zaynagetdinov R, Sherrill TP, Kendall PL, Segal BH, Weller KP, Tighe RM (2013). Identification of myeloid cell subsets in murine lungs using flow cytometry. Am J Respir Cell Mol.

[22] Locke LW, Schlesinger LS, Crouser ED (2020). Current sarcoidosis models and the importance of focusing on the granuloma. Front Immunol.

[23] Manna SK, Aggarwal BB (2008). α-Melanocyte-stimulating hormone inhibits the nuclear transcription factor NF-κB activation induced by various inflammatory agents. J Immunol.

[24] Singh M, Mukhopadhyay K (2014). Alpha-melanocyte stimulating hormone: an emerging anti-inflammatory antimicrobial peptide. BioMed Res Int.

[25] Cao W, Li M, Wu T, Feng F, Feng T, Xu Y (2017). αMSH prevents ROS-induced apoptosis by inhibiting Foxo1/mTORC2 in mice adipose tissue. Oncotarget.

[26] Wen AY, Sakamoto KM, Miller LS (2010). The role of the transcription factor CREB in immune function. J Immunol.

[27] Cinetto F, Scarpa R, Dell’Edera A, Jones MG (2020). Immunology of sarcoidosis: old companions, new relationships. Curr Opin Pulm Med.

[28] Giacomini E, Iona E, Ferroni L, Miettinen M, Fattorini L, Orefici G (2001). Infection of human macrophages and dendritic cells with Mycobacterium tuberculosis induces a differential cytokine gene expression that modulates T cell response. J Immunol.

[29] Cooper AM, Magram J, Ferrante J, Orme IM (1997). Interleukin 12 (IL-12) is crucial to the development of protective immunity in mice intravenously infected with Mycobacterium tuberculosis. J Exp Med.

[30] Akahoshi M, Ishihara M, Remus N, Uno K, Miyake K, Hirota T (2004). Association between IFNA genotype and the risk of sarcoidosis. Hum Genet.

[31] Sweiss NJ, Zhang W, Franek BS, Kariuki SN, Moller DR, Patterson KC (2011). Linkage of type I interferon activity and TNF-alpha levels in serum with sarcoidosis manifestations and ancestry. PLoS ONE.

[32] Timmermans WMC, van Laar JAM, van Hagen PM, van Zelm MC (2016). Immunopathogenesis of granulomas in chronic autoinflammatory diseases. Clin Transl Immunol.

[33] Sarkar A, Sreenivasan Y, Manna SK (2003). α-Melanocyte-stimulating hormone inhibits lipopolysaccharide-induced biological responses by downregulating CD14 from macrophages. FEBS Lett.

[34] Taylor A (2005). The immunomodulating neuropeptide alpha-melanocyte-stimulating hormone (α-MSH) suppresses LPS-stimulated TLR4 with IRAK-M in macrophages. J Neuroimmunol.

[35] Yoon S-W, Goh S-H, Chun J-S, Cho E-W, Lee M-K, Kim K-L (2003). α-Melanocyte-stimulating hormone inhibits lipopolysaccharide-induced tumor necrosis factor-α production in leukocytes by modulating protein kinase A, p38 kinase, and nuclear factor κB signaling pathways. J Biol Chem.

[36] Mandrika I, Muceniece R, Wikberg JE (2001). Effects of melanocortin peptides on lipopolysaccharide/interferon-gamma-induced NF-kappaB DNA binding and nitric oxide production in macrophage-like RAW 2647 cells: Evidence for dual mechanisms of action. Biochem Pharmacol.

[37] Liu Y, Promeneur D, Rojek A, Kumar N, Frøkiær J, Nielsen S (2007). Aquaporin 9 is the major pathway for glycerol uptake by mouse erythrocytes, with implications for malarial virulence. PNAS.

[38] Dunlock V (2020). Tetraspanin CD53: an overlooked regulator of immune cell function. Med Microbiol Immunol.

[39] Maler MD, Nielsen PJ, Stichling N, Cohen I, Ruzsics Z, Wood C (2017). Key role of the scavenger receptor MARCO in mediating adenovirus infection and subsequent innate responses of macrophages. MBio.

[40] Kurumagawa T, Seki S, Kobayashi H, Koike Y, Kanoh S, Hiraide H (2010). Characterization of bronchoalveolar lavage T cell subsets in sarcoidosis based on CD57, CD4 and CD8. Clin Exp Immunol.

[41] Brighenti S, Andersson J (2010). Induction and regulation of CD8+ cytolytic T cells in human tuberculosis and HIV infection. Biochem Biophys Res Commun.

[42] Parasa VR, Forsslund H, Enger T, Lorenz D, Kullberg S, Eklund A (2018). Enhanced CD8+ cytolytic T cell responses in the peripheral circulation of patients with sarcoidosis and non-Löfgren's disease. Respir Med.

