# Supplementary material for: Anti-inflammatory Properties of the Alpha-Melanocyte-Stimulating Hormone in Models of Granulomatous Inflammation
Source: Lung. 2022 Jun 18;200(4):463–72. doi: 10.1007/s00408-022-00546-x (PMC9360058; doi:10.1007/s00408-022-00546-x)
Supplement: Supplementary file 1 — Supplementary file. (DOCX 4859 KB) [file 408_2022_546_MOESM1_ESM.docx]

**Supplementary Information**

**Anti-inflammatory properties of the alpha-melanocyte -stimulating hormone in models of granulomatous inflammation**

Abdolrazagh Hashemi Shahraki **^1^**, Runxia Tian**^2^**, Chongxu Zhang^2^, Nevis L Fregien^2^, Pablo Bejarano^3^, and Mehdi Mirsaeidi **^1*^**

1. Division of Pulmonary, Critical Care and Sleep, College of Medicine-Jacksonville, University of Florida, Florida, FL, USA
2. Department of Cell Biology, University of Miami, Miami, FL, USA
3. Department of Pathology, Cleveland Clinic, Weston, FL

Corresponding author: Mehdi Mirsaeidi MD, 655 West 11th Street, Jacksonville, Florida, USA, 32209; m.mirsaeidi@ufl.edu

**Supplementary Table 1:** Demographic data and smoking history of lung donors used in this study are presented.

| NBECs IDs | Gender | Age | Smoking | Race |
| --- | --- | --- | --- | --- |
| 1 | Male | 67 | No | Latino |
| 2 | Male | 36 | EX | EA |
| 3 | Male | 22 | Yes | AA |
| 4 | Female | 16 | NA | Latino |
| 5 | Female | 57 | No | EA |

NA: not available, EX: ex-smoker, EA: European American, AA: African American

**Supplementary Figure 1:** A LOMM model developed from doners NBECs and lung microvascular endothelial cells. (a) LOMM with the red arrow pointing to membrane and communicating vesicles into intracanal the polycarbonate membrane 0.4 μm pore magnification X4000, Exposure 3000 (ms). (b) NBECs cells and red arrow pointing to the cilia, magnification magnification X400, exposure 3000 (ms). (c) shows LOMM displaying endothelial cells (arrows), magnification X1500, exposure 3000 (ms).


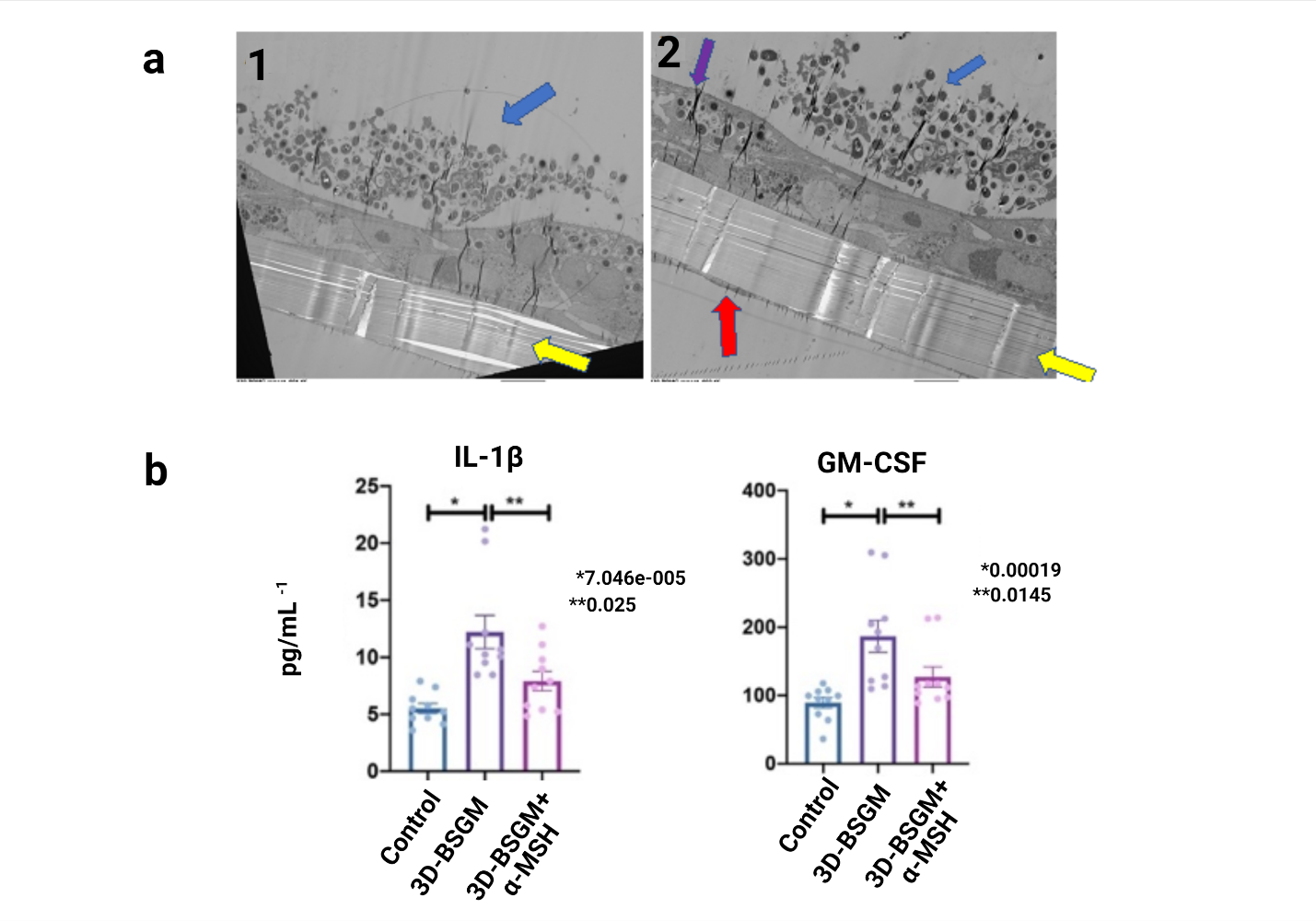


**Supplementary Figure 2:** Electron microscopic images of combined granuloma and LOMM called 3D-BSGM. 1. Magnification 400X 2. magnification 500X. (a) The Blue arrow shows granuloma including lymphocytes and macrophages, the yellow arrow shows membrane, the red arrow shows endothelial cells, and the purple arrow shows NBECs. (b) IL-1β and GM-CSF concentrations in the 3D-BSGM with and without α-MSH treatment relative to the control LOMM+ unchallenged PBMCs. For this experiment, 10 3D-BSGM were included in each group (2 replicates x NBECs from 5 donors x 3 groups = 30 experiments). Significant variations are highlighted for each plot.

**Supplementary Figure 3:** Protein–protein interaction network analysis of dysregulated genes in the integrated granuloma and 3D-BSGM model treated with α-MSH using STRING. The 68 top downregulated coding genes were entered into the Search Tool for the Retrieval of Interacting Genes (STRING) database for protein–protein interaction (PPI) network analysis.

**Supplementary Figure 4:** Shows biologic process in the integrated granuloma and 3D-BSGM model treated with α-MSH


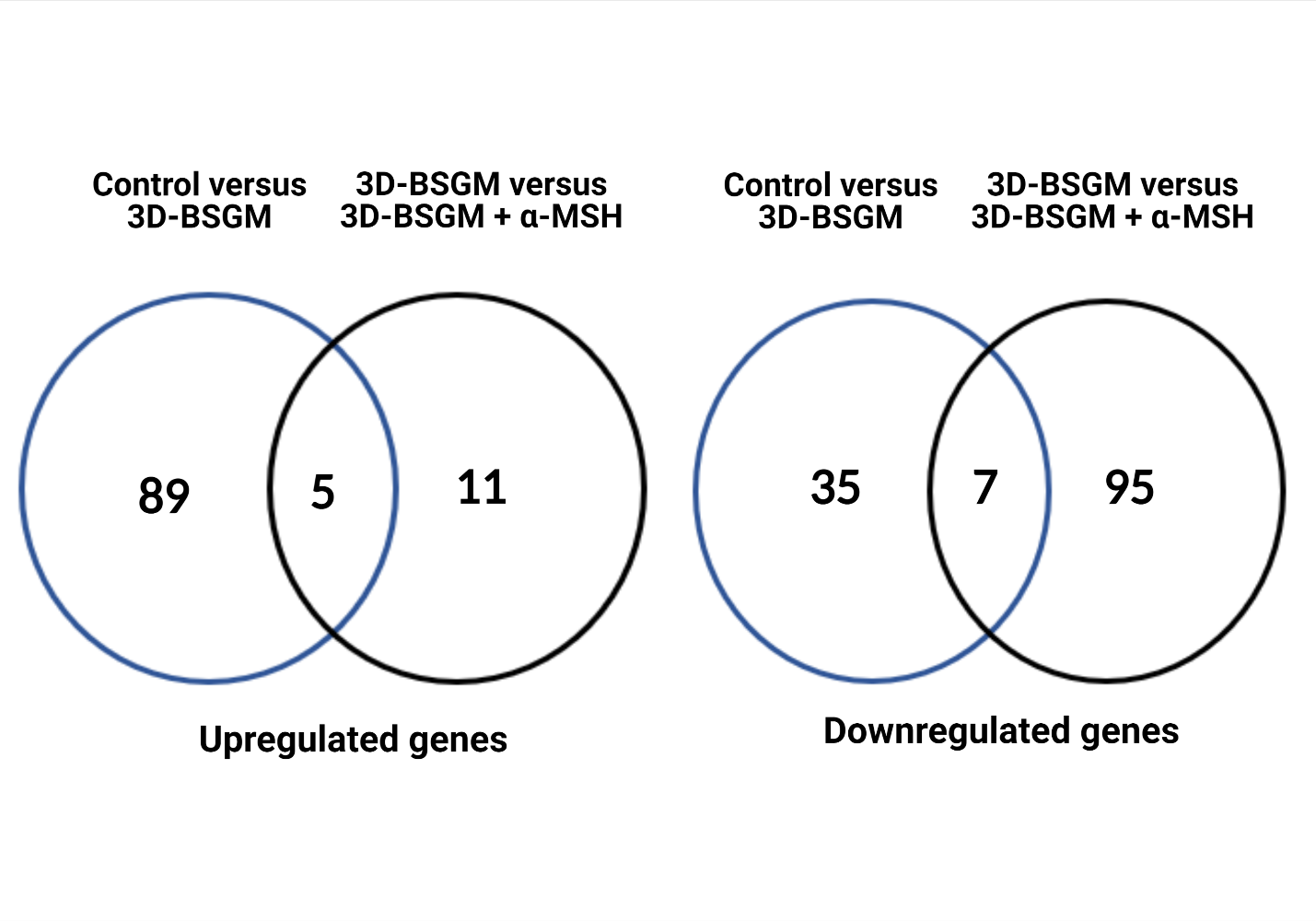


**Supplementary Figure 5:** Identification of altered genes with potential gene interactions in three different models (control, 3D-BSGM, 3D-BSGM+α-MSH). The Venn diagram analysis of predicted targets of dysregulated mRNAs identified 5 upregulated genes and 7 downregulated genes with potential interactions.

**
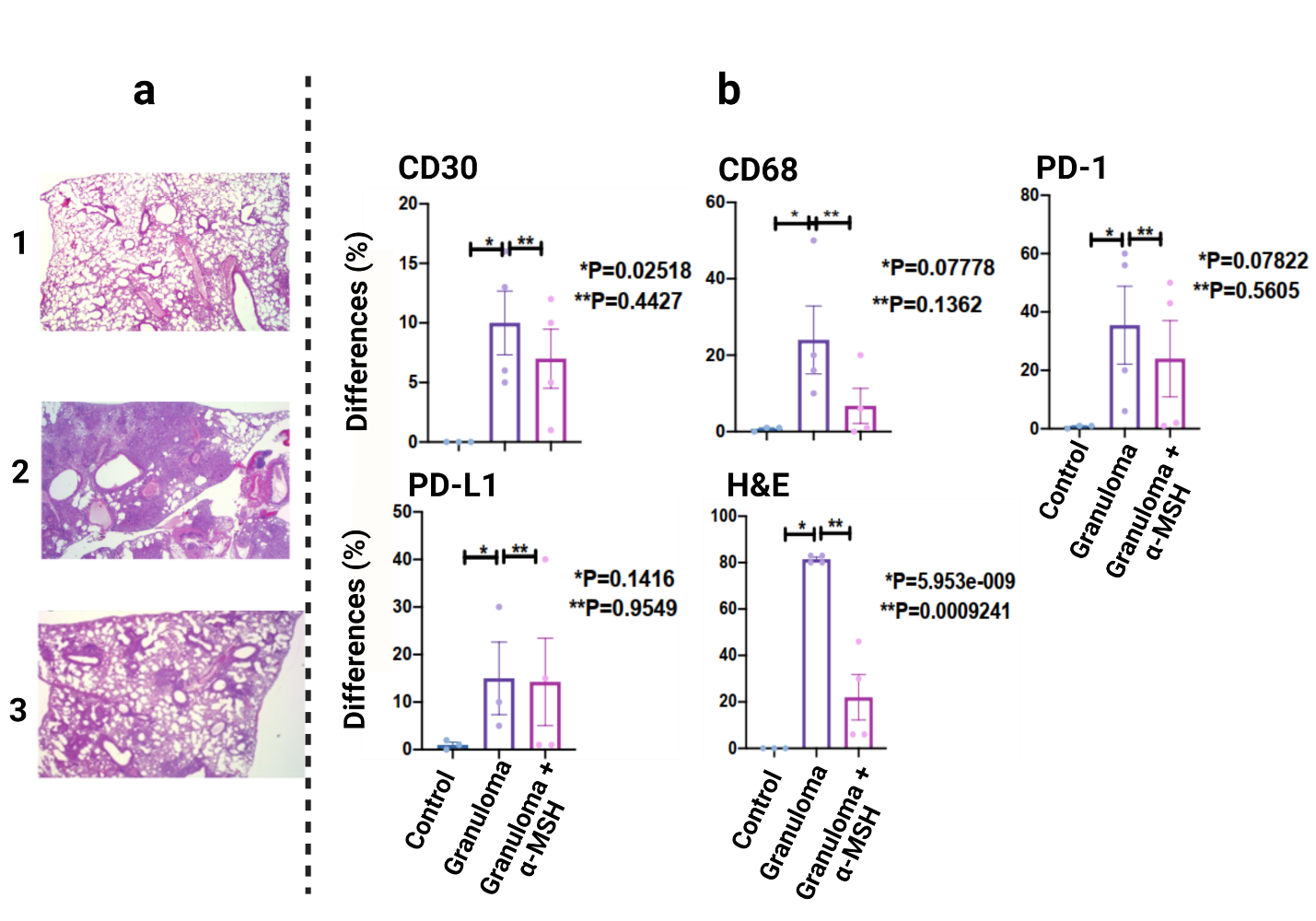
**

**Supplementary Figure 6.** Lung inflammation and markers in sarcoidosis mice model. **(a)** Lung inflammation evaluation with H&E staining; 1) control; only received saline, 2) granuloma group; received MAB microparticles and saline 3) granuloma + α-MSH group; received MAB microparticles and α-MSH. **(b)** Bar graphs also show the changes of different markers (CD30, CD68, PD-1 and PD-L1) in mice lungs according to their treatment. Each model had four mice. For H&E graph, the percentage of inflammation was scored by a pathologist in different groups. Significant variations are highlighted for each plot.
